# Geopolymer Composites—In Environmentally Friendly Aspects

**DOI:** 10.3390/gels9030196

**Published:** 2023-03-04

**Authors:** János Kóth, Katalin Sinkó

**Affiliations:** Institute of Chemistry, Eötvös Lóránd University, H-1117 Budapest, Hungary

**Keywords:** gel, composite, geopolymer, NMR, XRD, compressive strength

## Abstract

In the present work, a new, low energy consumption sol–gel synthesis route has been developed for geopolymer composites. Instead of the typically published 0.1–1.0 Al/Si molar ratios, the efforts of this study concentrated on the realization of >2.5 Al/Si molar ratios in the composite systems. The higher Al molar ratio significantly improves the mechanical properties. A very important aim was also the recycling of industrial waste materials with attention to environmentally friendly requirements. The very dangerous, toxic red mud as a waste product of aluminum industrial fabrication was selected for reclamation. The structural investigation was carried out by ^27^Al MAS NMR, XRD, and thermal analysis. The structural examination has unambiguously proven the composite phases in both gel and solid systems. The characterization of composites was performed with mechanical strength and water solubility measurements.

## 1. Introduction

Nowadays, many fields of industry require more environmentally friendly alternatives to traditional materials and technologies. The construction industry pays attention to the development and application of geopolymers. Geopolymer is a common term for an aluminosilicate polymer system, which generally includes alkaline, or alkaline earth metal ions [1]. In addition, the geopolymers can be composed of different industrial wastes. Two types of geopolymers can be classified; natural and synthetic products. The most natural geopolymers are clay minerals, such as kaolinite or illite. The structure of geopolymers is generally amorphous or semi-amorphous. The aluminosilicate network consists of SiO_4_^−^ and AlO_4_^−^ tetrahedrons connected by sharing the oxygen atom. The structural holes are filled by cations, such as Na^+^, K^+^ or Ca^2+^, which can stabilize the structure by means of balancing the negative charge of the AlO_4_^−^ ion [2,3]. The chemical composition of geopolymers can be characterized by the following simple empiric formula: M_n_(-(SiO_2_)_z_-AlO_2_)_n_·wH_2_O, where M is a cation with positive charge and *n* is the degree of polycondensation. There are a lot of examples of industrial waste which can be combined with synthetic or neutral aluminosilicate; fly ash, furnace slag, glass waste, sludge, etc. [4,5,6,7]. Red mud is a bauxite residue, which is a waste product of aluminum industrial fabrication using the Bayer process. There are few examples for the application of red mud in the geopolymer procedures [8,9].

The low energy consumption of the preparation methods, the cheap raw materials, and the potential recycling of industrial waste make geopolymers an attractive, environmentally friendly new class of materials for various industrial applications [10]. They are excellent protective coating materials, due to their fire and heat resistance; furthermore, they have low water permeability and low deformation [11]. They are applied as fire resistant coating for wallboards or replacement for cement [12,13,14]. Geopolymer composites are also produced to replace the plastic components in airplanes in order to avoid released poisonous and flammable gases [15]. Due to their long-term chemical resistance, they can be used to store radioactive or toxic materials, which cannot escape from the geopolymeric matrix [16]. The geopolymers can also be used in therapeutic fields, e.g., for bone tissue engineering [17]. 

The synthesis of geopolymers is generally called geopolymerization [18]. The precursors of geopolymerization may be synthetic molecules (inorganic salts such as Ca(NO_3_)_2_, Al(NO_3_)_3_, and Na_2_SiO_3,_ or organic salts, e.g., metal alkoxides [18,19]) and natural materials (clay minerals, perlite, feldspar [20]). Furthermore, even industrial wastes may be taken into account [4,5,6,7,21]. In classic geopolymerization, strong bases (NaOH or KOH) must be used to produce an aqueous solution from the starting materials [18,22]. The chain-like aluminosilicates (e.g., augite) can be dissolved more easily in basic solution than the silicates with 3D structures (e.g., sodalite) [23]. The basic process of geopolymerization is condensation. The silicates (e.g., Al silicates, [M_z_(AlO_2_)_x_(SiO_2_)_y_] or Na silicate, Na_2_SiO_3_) react with the Al-containing components (e.g., Al salts, AlOOH, Al_2_O_3_·H_2_O) in basic solution and produce dimers and trimers, and then they react with larger oligomers by means of Al–O–Si or Si–O–Si bonds. The condensation is carried out by powder or melting technology at higher temperature [18,24]. 

Nowadays, besides the classic geopolymerization method, several new synthesis routes have been developed. New preparation techniques include co-precipitation [25] and hydrothermal methods [26]; and the most promising route is the low-energy-consuming sol–gel technique [27,28,29,30,31,32]. The aluminosilicate materials prepared by the sol–gel method can be characterized by high purity and homogeneity, good uniformity, and good reactivity to other components. The typical starting materials in sol–gel chemistry are alkoxides (Al *iso*propoxide, tetraethoxysilane, TEOS) and ethanol as a solvent [27,28,29,30,31]. The fundamental reactions are hydrolysis and condensation, resulting in 3D aluminosilicate networks. There are few exemplars for the application of Al nitrate besides TEOS in the sol–gel technique [32,33]. Sometimes, the sol–gel method is used only for the separate preparation of SiO_2_ and Al_2_O_3_/AlOOH components, and then the particles are subjected to react to each other in a heating step [34,35].

In the present work, special attention was paid to development of a new sol–gel synthesis method for geopolymer composites. The published research syntheses or industrial fabrications produce geopolymers with 0.1–1.0 Al/Si molar ratios. One of our aims was to create geopolymer systems with >2.5 Al/Si molar ratios and to investigate their structures and mechanical properties. Another aim was the recycling of industrial waste materials in respect of environmentally friendly requirements. The very dangerous, toxic red mud (bauxite) mining waste was selected for reclamation. The structural investigation was carried out by ^27^Al MAS NMR, XRD, and thermal analysis. The characterization of composites was based on mechanical strength and water solubility measurements. 

## 2. Results and Discussion

### 2.1. Synthetic Geopolymer Synthesis

The main reason for the application of AlAc ⌠Al(OH)(OOCCH_3_)_2_⌡ is its environmentally friendly character, owing to the decomposition of acetate ions to CO_2_ and H_2_O molecules during gelation and heating. Further advantages of AlAc are its low cost and controllable hydrolysis. The application of water glass (sodium metasilicate, Na_2_SiO_3_) was motivated by its fast and intensive reaction with Al(III) ions and low price. The process is based on sol–gel chemistry results in a new type of aluminosilicate composite wet gel. The structure and morphology of the dried gel is predominantly determined by the Al/Si molar ratio. At ratios <2, an amorphous weak monolithic gel forms. The gel is easily broken by drying at ~100 °C. At >4 Al/Si molar ratios, monolithic gel structures cannot be obtained, as a phase separation occurs. Homogeneous monolithic gel systems with very good mechanical properties can be produced by using 2.5–3.5 Al/Si molar ratios. Heating the gels leads to the formation of hard ceramic monoliths [36]. These aluminosilicate products possess a new, unique composite structure. The composite structure is built up from an amorphous aluminosilicate network and an Al-containing crystalline as a reinforcement phase [36]. Both the gel network and the nano phase develop together during the gelation from the starting solution. During the formation of the gel skeleton, the size of the crystallite remains in nano size. The 3D gel structure is not destroyed by the effect of crystallization due to the nanocrystalline phase. Below 300 °C, hydrated aluminum oxide may be identified as being in an Al-containing nanocrystalline phase, while above 500 °C, it is in a zeolite-type sodium aluminosilicate (NaAlSiO_4_·H_2_O) phase [36]. The pure synthetic geopolymer was used for comparison in this research work.

### 2.2. Synthesis of Geopolymer Composites from Industrial Wastes

The addition of red mud or red mud with reduced iron and rare earth metal oxides (rmr) to the basic starting system of two synthetic precursors (AlAc, Na silicate) leads to the formation of a colloid solution with some red-colored precipitates. Removing the main part of the water content by distillation, the colloid solution becomes a slurry and then a highly opaque, compact, gel-like system by drying at 100 °C and at atmospheric pressure. A further heat treatment at a higher temperature (>400 °C) induces the formation of porous solid bulk products. A very important achievement of this synthesis is that it reliably produces intact monolith structures without cracking while going through fast, atmospheric heat treatment. Thus, a significant difficulty, i.e., the cracking of the wet gels during the heating processes, can be avoided. The fast, open drying without cracking of the gels is a unique process in the sol–gel technique. 

### 2.3. Structures of Geopolymer Composites

**Figure 1:**
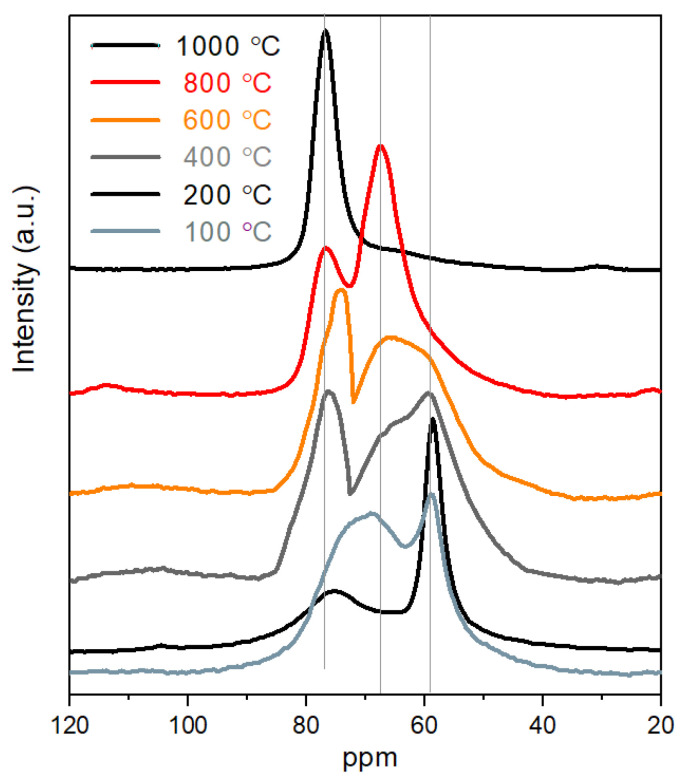
The ^27^Al MAS NMR spectra of synthetic geopolymer vs. temperature of heat treatment.

The composite structure of synthetic geopolymers has been proven by ^27^Al MAS NMR (Figure 1). The composites are built up from a 3D amorphous aluminosilicate network and a crystalline Al-containing phase embedded in the amorphous network. The NMR signals represent two types of Al content. The Al NMR peak at approximately 60 ppm can be attributed to tetrahedrally incorporated Al atoms (AlO_4_^−^ units) into the silica systems (amorphous aluminosilicate network and ordered zeolite-like structure) [36,37]. The other Al-containing component can be observed at 74–75 ppm representing a dispersed Al-containing phase (disordered Al oxide/hydroxide, NaAlSiO_4_) [38,39,40,41,42]. Above 300 °C, an intensive phase transformation occurs; a new broad signal indicates it at 68 ppm. The amorphous aluminosilicate network and zeolite-like structure turns into another ordered type of zeolite-like phase (as represented in the XRD measurements). The zeolites possess a polymeric Si–O–Al framework, similar to geopolymers, but with a predominantly crystalline structure. The aluminum content is also basically tetrahedrally coordinated in the zeolite-type phases [43,44]. Between 600 °C and 800 °C, this signal becomes stronger. Above 600 °C, a new phase starts to develop—the θ-Al_2_O_3_ phase—derived partly from the decomposition of the aluminosilicate phase [38,45]. Its other source is γ-Al_2_O_3_, which evolves at above 300 °C. Thus, the temperature of the heat treatment of the geopolymer may be a maximum 600 °C.

**Figure 2:**
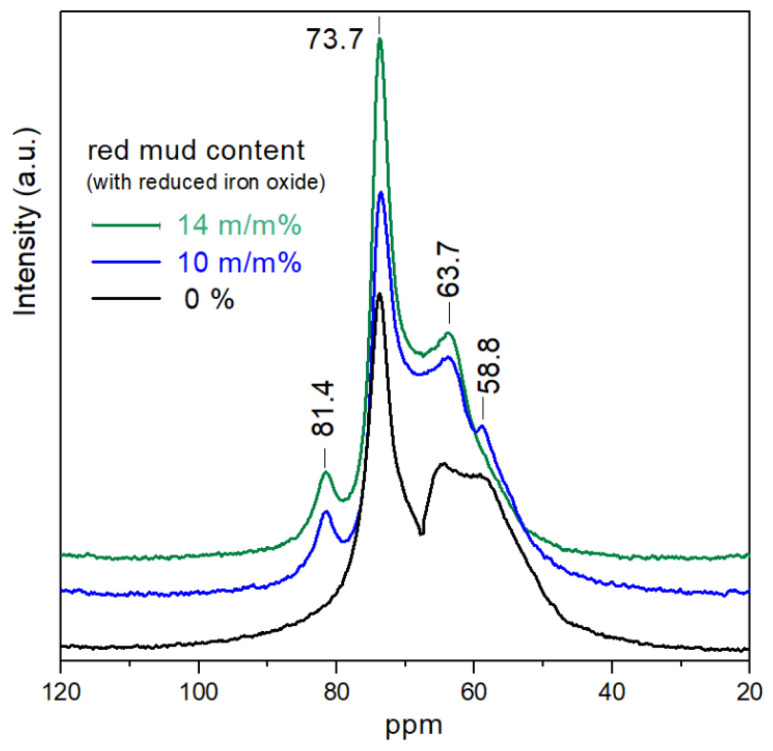
^27^Al MAS NMR spectra of samples heat-treated at 600 °C vs. rmr content.

Figure 2 represents Al NMR spectra of samples with different—reduced iron and rare earth metal oxide-containing—red mud (rmr) content. The samples with original red mud content cannot be used for Al NMR investigation due to the strong ferromagnetic effect of iron oxide. 

A new phase can be observed on the NMR curve performed with the rmr-containing samples at 81.4 ppm. This signal does not appear on the curve of the sample without red mud. The peak can be attributed to the calcium aluminosilicate phase [42,46,47]. The signal of θ-Al_2_O_3_ and NaAlSiO_4_ phases become more intense and sharper owing to the Al-containing phases of red mud. The signal of amorphous aluminosilicate network cannot be identified in the sample of higher rmr content. Zeolite-like systems form instead of pure Al silicates because the Al and Si ions react with the increased number of alkali and alkaline earth ions. 

**Figure 3:**
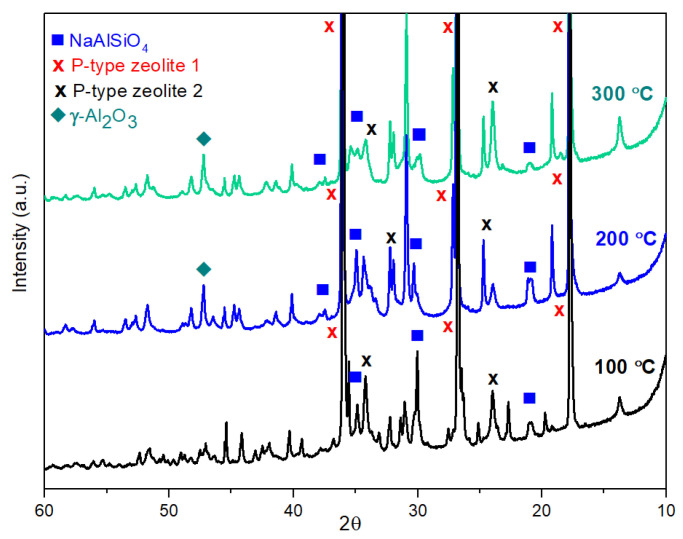
X-ray powder diffraction spectra of synthetic geopolymer samples heat-treated at 100 °C, 200 °C, and 300 °C.

**Figure 4:**
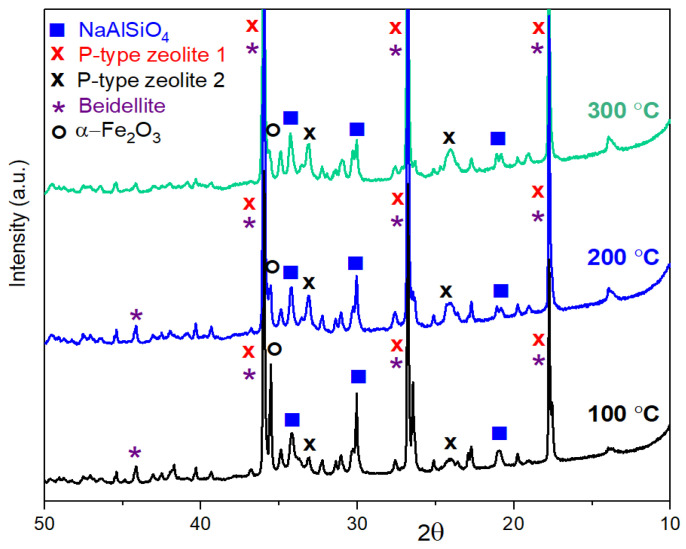
X-ray powder diffraction spectra of samples containing 33% red mud heat-treated at 100 °C, 200 °C, and 300 °C.

**Figure 5:**
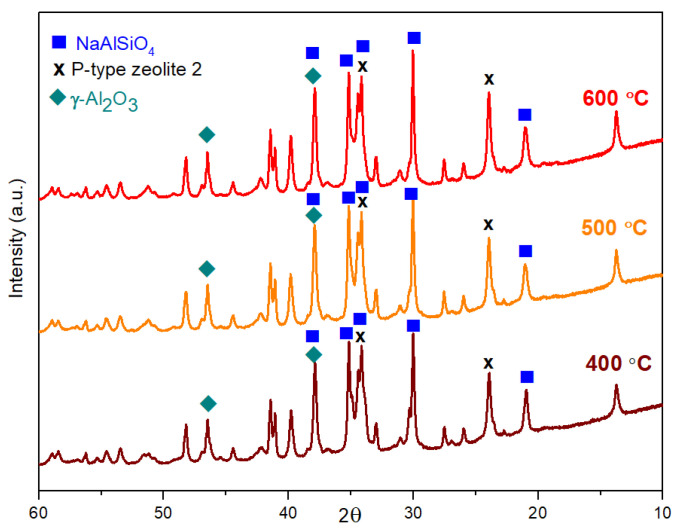
X-ray powder diffraction spectra of synthetic geopolymer samples heat-treated at 400 °C, 500 °C, and 600 °C.

**Figure 6:**
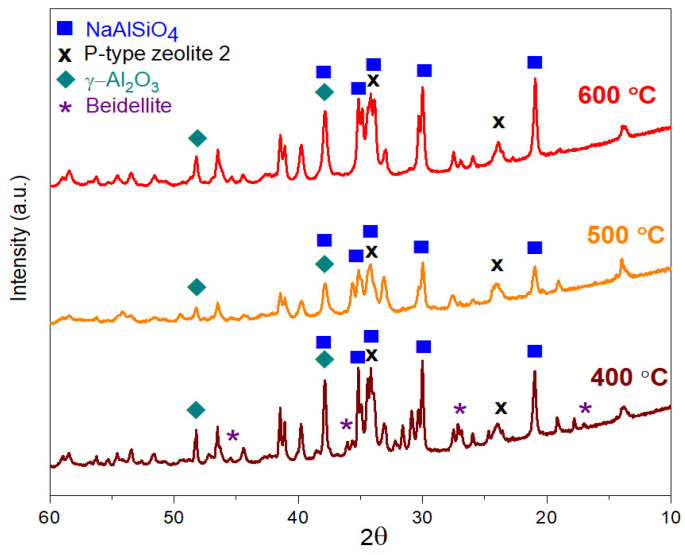
X-ray powder diffraction spectra of samples containing 33% red mud heat-treated at 400 °C, 500 °C, and 600 °C.

The XRD measurements verify “p-type zeolite 1” phase (JCPDS card #01-073-9491) in very high intensity compared with the other phases in both geopolymer composite samples with or without red mud content. This significant phase remains until 300 °C (Figure 3 and Figure 4). Above 300 °C, this phase disappears; it mostly turns into NaAlSiO_4_ (JCPDS card #00-042-0217) and “p-type zeolite 2” (JCPDS card #00-011-0221) phases and in smaller volume into γ-Al_2_O_3_ (JCPDS card #00-10-0425) (Figure 5 and Figure 6). The crystalline phase of γ-Al_2_O_3_ is negligible in the samples treated up to 300 °C in both types of composites. The phases of γ-Al_2_O_3_ and NaAlSiO_4_ remain until 600 °C in the composites. Above 600 °C, γ-Al_2_O_3_ becomes the θ-Al_2_O_3_ phase (JCPDS card #11-0517) as proven by Al NMR. The calcium aluminosilicate phase, as indicated by the NMR, may be identified by XRD as a beidellite phase. It only appears between 100 °C and 400 °C. The beidellite phase is difficult to identify unambiguously since its main peaks are mostly overlapped by the zeolite phases. The important phase of red mud, the α-Fe_2_O_3_ phase, can also be identified in the diffractograms only between 100 °C and 400 °C. The red mud contains α-Fe_2_O_3_ in 32–37 *w*/*w*%. 

**Figure 7:**
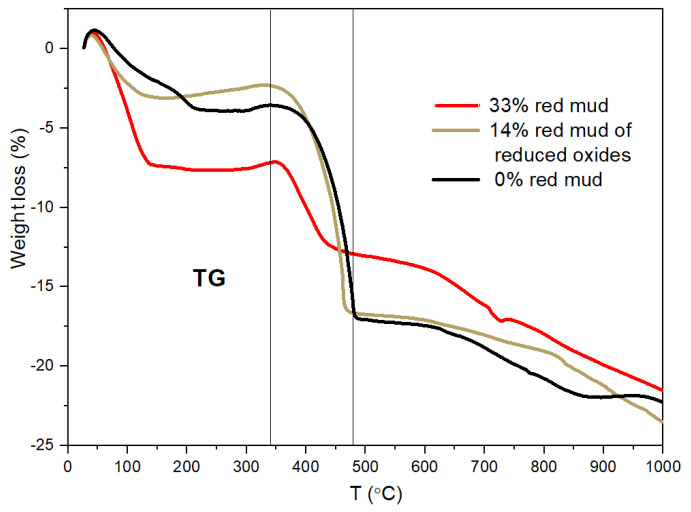
Thermogravimetry measurements of composites vs. red mud content.

**Figure 8:**
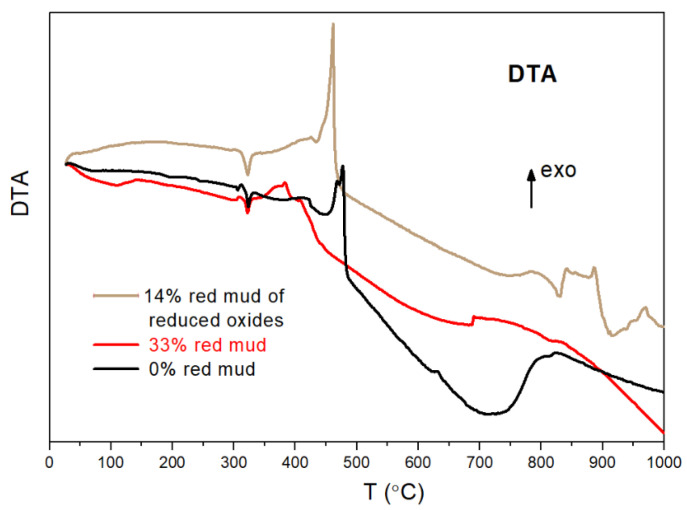
Differential thermal analysis of composites vs. red mud content.

The intensive changes above 300 °C have also been confirmed by thermal analysis (Figure 7 and Figure 8). A two-step weight loss can be observed up to 480 °C. The first weight loss can be attributed to remaining solvent content after the drying process at 80 °C. The biggest weight loss starts at ~340 °C and finishes at 480 °C. The weight loss of the composite samples during this step is 10–15% depending on the chemical composition. The main part of weight loss in this range can be associated with the escape of acetate ions. It has been proven that weight loss is lowest (~10%) in the sample with 33 *w*/*w*% red mud and the lowest acetate content. On the DTA curves, there are exothermic peaks at 460–480 °C (Figure 8). These exothermic peaks can be attributed to the combustion of acetate ions and to a crystalline phase transformation, e.g., γ-Al_2_O_3_. 

All structural examinations, i.e., ^27^Al MAS NMR, XRD, and TA, have proven significant structural changes above 300 °C. The phases of p-type zeolite 1, beidellite, disordered Al oxide/hydroxide, and α-Fe_2_O_3_ disappear due to the various processes. The processes may be chemical reactions between the components, resulting in disordered (various silicates) and crystalline products (p-type zeolite 2, NaAlSiO_4_). The other type of process is crystallization (γ-Al_2_O_3_ and then θ-Al_2_O_3_). 

### 2.4. Characterization of Geopolymer Composites

**Figure 9:**
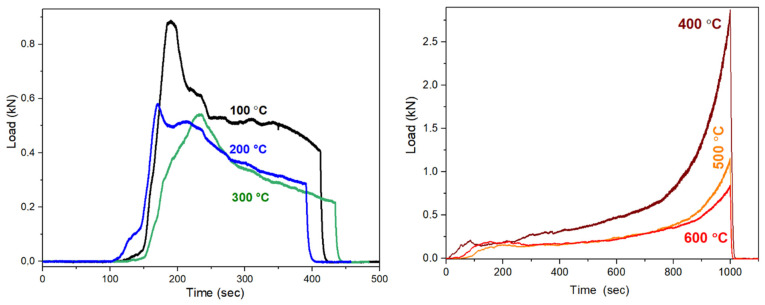
The load vs. time curves of the MTS compressive strength measurement of the synthetic composite heated at 100–300 °C and 400–600 °C.

**Figure 10:**
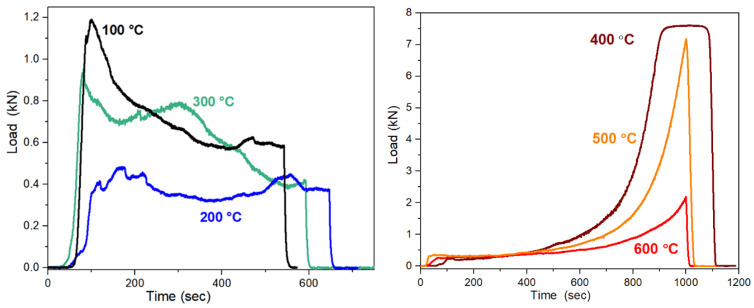
The load vs. time curves of the MTS compressive strength measurement of the red mud-containing composite heated at 100–300 °C and 400–600 °C.

The behavior of composites under the load was measured by means of the MTS (Material Testing System 810) technique. The compressive strength was investigated as a function of heating temperature on both synthetic and red mud-containing samples (Figure 9 and Figure 10). The results of the tests are collected in Table 1. The load—time curves for synthetic geopolymers between 100 °C and 300 °C show a peak and then a gradual but not monotone decrease as a function of load time. The sharper peak presents a brittle fracture process. The peak together with a slope on the load curve indicate a dual behavior; it proves the presence of at least one brittle component and one tough material component in the samples. The dual behavior confirms the composite structure; the crystalline phase provides the brittle component, and the 3D aluminosilicate network provides the tough, flexible component. Comparing the curves as a function of the heating temperature, the maximum load of the curve becomes lower and the first peak of the brittle component fractures becomes wider. Thus, the aluminosilicate network becomes more ordered at a higher temperature. Above 300 °C, the peak at the beginning of load curves is missing and an increase in the compressive strength can be observed by the passing of time. The missing peak denotes the disappearance of the brittle phase. The evaluation of the load curves supports the structural changes, as also determined by Al NMR and XRD. However, the maximum load becomes lower if the treatment temperature is higher due to the conversion of the tough, flexible 3D aluminosilicate network.

At the measurement of the red mud-containing samples, identical tendencies can be observed as in the synthetic samples (Figure 10). Although the effect of the heat treatment temperature is similar, much higher load values could be detected than in the synthetic samples. The loadability of the red mud-containing composites treated at ≤300 °C is larger by 20–30%; that of the composites treated at ≥400 °C is 3–6 times larger (Table 1). On the load curves of the composites treated at ≤300 °C, a second but smaller maximum can be observed refereeing more crystalline phases than in the synthetic geopolymer composite. The yield point in the case of most samples was between 0.4 (1.3 MPa) and 3 kN (9.5 MPa), while it was much higher in the case of the 500 °C and 400 °C samples, resulting in comparable compressive strength to cement. The maximum load at the samples treated at 400 °C is 25 MPa; the typical compressive strength of the cement is between 15 and 33 MPa. 

The data of Table 1 verify that the red mud content strongly improves the mechanical properties of the geopolymer composites. The best values belong to the composites heat-treated at 400–500 °C. 

Regarding the application of the geopolymer composites, water solubility is a relevant parameter. The determination of dissolved ions was carried out by the inductively coupled plasma mass spectrometry (ICP-MS) technique. The most important results are summarized in Table 2. The highest concentration of filtrates in the water solubility experiments connects to sodium ions in both composites. The Na ions are derived from the NaOH solution used to dissolve the Al acetate. The main part of an Na ion reacts in chemical processes, but its small part remains unreacted and water-soluble. The aluminum content possesses the second highest concentration, but no high concentration resulted from the unreacted Al acetate. The number of water-soluble Al ions is greater in the composite of red mud than in the synthetic samples due to the reactivity of Al content in the red mud. This Al content can replace the Al acetate in the reactions. 

Water solubility strongly depends on the heat treatment temperature. Water solubility drastically reduces at 400 °C in the case of synthetic geopolymers and at 300 °C for the red mud-containing composites. Above 300–400 °C, water solubility can be neglected (< 5*w*/*w*%) in the composites. 

## 3. Conclusions

The objective of this work was to develop a new sol–gel synthesis for geopolymer composites. One of the aims was to prepare geopolymer systems with >2.5 Al/Si molar ratios and good mechanical properties. The published research and industrial syntheses produce geopolymers with 0.1–1.0 Al/Si molar ratios. By using 2.5–3.5 Al/Si molar ratios, homogeneous monolithic gel systems with good mechanical properties could be achieved. These aluminosilicate products possess a new, unique composite structure. The composite structure is built up from an amorphous aluminosilicate network and an Al-containing component as a dispersed reinforcement crystalline phase. The aluminosilicate gel systems can be quickly dried under ambient pressure without cracking, which is a unique feature of the drying process in the sol–gel technique. This unique drying process, the low energy consumption, and the special composite product all provide the important novelty for this developed sol–gel procedure.

The other aim was the recycling of industrial wastes whereby fulfilling environmentally friendly requirements. The very dangerous, toxic red mud as a waste product of aluminum industrial fabrication was selected in this work. 

The structural investigations, i.e., ^27^Al MAS NMR, XRD, and TA analysis, have proven a composite structure in both synthetic and red mud-containing geopolymers with an amorphous or crystalline aluminosilicate phase with a more or less 3D framework and a high Al-containing dispersed crystalline phase. Significant structural changes occur above 300 °C. Below 300 °C, the “p-type zeolite 1” phase is the dominant crystalline phase. Above 300 °C, the phases of p-type zeolite 1, beidellite, disordered Al oxide/hydroxide, and α-Fe_2_O_3_ disappear due to the various reactions. The reactions result in disordered (various silicates) and crystalline products (p-type zeolite 2, NaAlSiO_4_). At 600 °C, a new phase develops—the θ-Al_2_O_3_ phase—derived from the decomposition of the aluminosilicate and the transformation of γ-Al_2_O_3_ phases. 

The compressive strength data verify that the red mud content strongly improves the mechanical properties of the geopolymer composites. The composites heat-treated at 400–500 °C possess the best values, which compare well with the value of cement. The dual behavior in the measurements of compressive strength also confirms the composite structure; the crystalline phase provides the brittle component, and the 3D aluminosilicate network provides the tough, flexible component. The water solubility of synthetic geopolymers drastically reduces at 400 °C; that of red mud-containing composites at 300 °C. By heat treatment above 300–400 °C, water solubility can be neglected in the composites. 

According to NMR and XRD examinations, the temperature of the heat treatment of the geopolymer may be a maximum 600 °C. Regarding the results of compressive strength as well, the optimal temperature of the heat treatment for geopolymer composites is 500 °C. 

## 4. Experimental Part

### 4.1. Synthesis

#### 4.1.1. Synthesis of Synthetic Geopolymers

A synthetic geopolymer was provided for comparison. Basic aluminum acetate (Al(OH)(OOCCH_3_)_2_, AlAc, Aldrich) and sodium silicate (Na_2_SiO_3_) with a 3:1 ratio in aqueous NaOH solution using pH = 14 was stirred at 60 °C. Basic Al acetate can only be dissolved in a strongly acidic (pH ≤ 1) or basic (pH > 11) medium. Using Na_2_SiO_3_ only, the basic medium may be relevant, avoiding the phase separation of (poly)silicic acid. In order to get hydrogels from the obtained aqueous suspension, the main part of the solvent had to be evaporated at 60 °C. The highly opaque monolith gels were dried at various temperatures.

#### 4.1.2. Synthesis of Geopolymer Composites from Industrial Wastes

The red mud and the red mud with reduced iron and rare earth metal oxides (rmr) industrial wastes were added to the starting systems after the mixture of two synthetic precursors (AlAc and Na silicate). The main components of the wastes and their mineral sources are represented in Table 3. The chemical compositions of the starting materials in the series also had to be fitted to a 3:1 Al/Si ratio. This requirement limited the maximum weight of waste that could be incorporated into the synthetic components. The maximum weight of the red mud was 33 ± 3%, observing the 3:1 Al/Si molar ratio. This amount was 14 ± 2% for the red mud with reduced iron and rare earth metal oxides content. The next step was the evaporation of the solvent at 100 °C. The final heat treatment was performed for 4 h at various temperatures. The optimal temperature proved to be approximately 500–600 °C. The experiments with rmr-containing samples mainly provided the Al MAS NMR spectroscopy measurements.

### 4.2. Investigation Methods

*^27^Al Magic Angle Spinning Nuclear Magnetic Resonance spectroscopy (^27^A MAS-NMR).* The ^27^Al MAS NMR spectra were recorded using a BRUKER DRX-500 NMR SPECTROMETER. The applied parameters were magnetic field 11,744T, 4 mm rotor, 99.6 MHz Larmor frequency, and a 54.74° angle to the magnetic field. The samples with high red mud content were not possible to measure due to their ferromagnetic effect. The ^27^Al chemical shifts were measured with respect to Al(H_2_O)_6_^3+^ as an external reference. The evaluation of the spectra composed of broad signals was realized by decomposition to Lorentz curves with WINFIT software.

*Powder X-ray diffraction measurements (XRD)* were performed by a Rigaku Smartlab X-ray diffractometer equipped with a 1.2 kW copper source (radiation wavelength: CuKα; λ = 0.15418 nm). The data were collected in the range 2Θ between 10° and 110° with a 1D silicon strip detector (D/Tex ultra-250) at a speed of 0.2°/min. The results were analyzed using databases, such as ICDD, and other data from the literature.

*Inductively coupled plasma mass spectrometry (ICP-MS)* investigation was carried out by an iCAP Q ICP-MS (Thermo Fisher Scientific) device, using quartz sample holders. The concentration values were calculated with 1000 times dilution and measured with the He collision mode. The set dwell time was 0.05 s, with 10 runs and 3 passes.

*Thermal analysis measurements* were conducted using Derivatograph-C System equipment between 25 °C and 1000 °C, under static air atmosphere, with 10 °C/minute heating rate. The weight change of the samples was followed with respect to the temperature by thermogravimetry (TG). TG was employed together with differential thermal analysis (DTA). The sample holder’s material and the reference material were both aluminum oxide.

*Compressive strength* of the samples was tested using a Material Testing System 810 device, in low pressure mode. The necessary force was measured to compress the samples with a constant rate of 0.01 mm/s. The samples were cylindrical shaped, with 20 mm diameter top and bottom sides, and with heights varying 13–16 mm.

## Figures and Tables

**Table 1:** The parameter of yield point and calculated hardness of the composites measured as a function of heat treatment.

Temperature of Heat Treatment (°C)	Yield Point Parameters of Synthetic Geopolymer Composites		Yield Point Parameters of Red Mud-Containing Composites	
	Maximal Load/kN	Compressive Strength/kPa	Maximal Load /kN	Compressive Strength/kPa
100	0.89	2.826	1.18	3.78
200	0.58	1.835	0.48	1.53
300	0.54	1.729	1.01	3.20
400	2.87	9.140	7.59	24.16
500	1.16	3.680	7.17	22.83
600	0.84	2.657	2.17	6.92

**Table 2:** The results of the ICP-MS investigation on the filtrates of water solubility measurements.

Ions	Monitored Isotope	*m* (cps dm^3^ μg^−1^)	Concentration (mg/dm^3^) ± SD	
			Red Mud Sample	Synthetic Sample
Na	23	2380	5636 ± 22	5261 ± 37
Al	27	508	262 ± 3	151 ± 2
K	39	956	3.20 ± 0.02	2.03 ± 0.6
V	51	22.182	1.93 ± 0.03	n.d.
Cr	52	34,523	0.74 ± 0.02	n.d.
Ca	44	437	<1.0	<1.0
Sc	45	656	<1.0	<1.0
Si	28	7689	<1.0	<1.0
Sb	121	27,577	<1.0	<1.0
Co	59	60,437	<0.5	<0.5
Mo	98	46,792	<0.5	<0.5
Sn	120	54,537	<0.5	<0.5
Cu	63	40,678	<0.3	<0.3
Rb	85	14,409	<0.3	<0.3
Sr	88	19,450	<0.3	<0.3
Ni	58	32,922	<0.1	<0.1
Fe	56	24,712	n.d.	n.d.

*m*: gradient of the linear calibration; SD: standard deviation; n.d.: non-detectable.

**Table 3:** The chemical components of red mud-based industrial wastes and their mineral sources.

Main Components	Red Mud (*w*/*w*%)	Red Mud with Reduced Oxides
Fe_2_O_3_ α-Fe_2_O_3_, FeO(OH)	32–37	11–12
Al_2_O_3_ γ-Al(OH)_3_, AlO(OH)	15–18	10–11
TiO_2_ rutile	5–8	5–6
SiO_2_ Na/Al/Ca mixed silicates	10–15	63–66
Na_2_O Na silicates	7–10	1–2
CaO CaCO_3_ (calcite)	4–7	4–4.5

## Data Availability

Not applicable.
